# Development of the Cook-Ed^TM^ Matrix to Guide Food and Cooking Skill Selection in Culinary Education Programs That Target Diet Quality and Health

**DOI:** 10.3390/nu14091778

**Published:** 2022-04-24

**Authors:** Roberta C. Asher, Tammie Jakstas, Fiona Lavelle, Julia A. Wolfson, Anna Rose, Tamara Bucher, Moira Dean, Kerith Duncanson, Klazine van der Horst, Sonja Schonberg, Joyce Slater, Leanne Compton, Roslyn Giglia, Sandra Fordyce-Voorham, Clare E. Collins, Vanessa A. Shrewsbury

**Affiliations:** 1 School of Health Sciences, College of Health, Medicine and Wellbeing, The University of Newcastle, Callaghan, NSW 2308, Australia; roberta.asher@uon.edu.au (R.C.A.); tammie.jakstas@uon.edu.au (T.J.); anna.rose@newcastle.edu.au (A.R.); clare.collins@newcastle.edu.au (C.E.C.); 2 Hunter Medical Research Institute, New Lambton Heights, NSW 2305, Australia; tamara.bucher@newcastle.edu.au (T.B.); kerith.duncanson@newcastle.edu.au (K.D.); 3 School of Biological Sciences, Institute for Global Food Security, Queen’s University Belfast, Belfast BT9 5DL, UK; flavelle01@qub.ac.uk (F.L.); moira.dean@qub.ac.uk (M.D.); 4 Department of International Health, Johns Hopkins Bloomberg School of Public Health, Baltimore, MD 21205, USA; jwolfso7@jhu.edu; 5 Department of Health Policy and Management, Johns Hopkins Bloomberg School of Public Health, Baltimore, MD 21205, USA; 6 Department of Health Management and Policy, School of Public Health, University of Michigan, Ann Arbor, MI 48109, USA; 7 School of Environmental and Life Sciences, College of Engineering, Science and Environment, The University of Newcastle, Callaghan, NSW 2308, Australia; 8 School of Medicine and Public Health, College of Health Medicine and Wellbeing, The University of Newcastle, Callaghan, NSW 2308, Australia; 9 School of Health Professions, Bern University of Applied Sciences, 3012 Bern, Switzerland; klazine.vanderhorst@bfh.ch (K.v.d.H.); sonja.schoenberg@bfh.ch (S.S.); 10 Department of Food and Human Sciences, Faculty of Agricultural and Food Sciences, University of Manitoba, Winnipeg, MB R3T 2N2, Canada; joyce.slater@umanitoba.ca; 11 Victorian Curriculum and Assessment Authority, Melbourne, VIC 3000, Australia; leanne.compton@education.vic.gov.au; 12 Foodbank Western Australia, Perth, WA 6105, Australia; roslyn.giglia@foodbankwa.org.au; 13 Mentone Girls’ Grammar School Melbourne, Melbourne, VIC 3194, Australia; sfordyce@mentonegirls.vic.edu.au

**Keywords:** food skills, cooking skills, education, culinary nutrition

## Abstract

Culinary education programs are generally designed to improve participants’ food and cooking skills, with or without consideration to influencing diet quality or health. No published methods exist to guide food and cooking skills’ content priorities within culinary education programs that target improved diet quality and health. To address this gap, an international team of cooking and nutrition education experts developed the Cooking Education (Cook-Ed^TM^) matrix. International food-based dietary guidelines were reviewed to determine common food groups. A six-section matrix was drafted including skill focus points for: (1) Kitchen safety, (2) Food safety, (3) General food skills, (4) Food group specific food skills, (5) General cooking skills, (6) Food group specific cooking skills. A modified e-Delphi method with three consultation rounds was used to reach consensus on the Cook-Ed^TM^ matrix structure, skill focus points included, and their order. The final Cook-Ed^TM^ matrix includes 117 skill focus points. The matrix guides program providers in selecting the most suitable skills to consider for their programs to improve dietary and health outcomes, while considering available resources, participant needs, and sustainable nutrition principles. Users can adapt the Cook-Ed^TM^ matrix to regional food-based dietary guidelines and food cultures.

## 1. Introduction

Cooking and food skills proficiency, and frequent consumption of home-prepared meals, are factors associated with higher diet quality, meaning dietary patterns more closely aligned with food-based dietary guidelines (FBDG) [1,2,3,4,5,6]. Many countries have created FBDGs to define dietary patterns associated with good health that also consider local cultural and geographical factors [7]. Across these country-specific FBDGs, the core nutrition principles consistently promote a dietary pattern with a high proportion and variety of plant-based whole foods such as vegetables, fruits, and wholegrains, with the addition of meat or meat alternatives, and often dairy or dairy alternatives [8,9,10,11]. However, the international literature indicates dietary intakes of most populations are not consistent with their nation’s respective FBDGs [12].

Culinary education programs that teach food skills* and cooking skills* for domestic applications consider food agency* to varying degrees and are delivered in a range of settings across education and health sectors (* defined in Box 1) [13,14,15,16,17]. These programs commonly report positive outcomes such as improvements in diet quality [13,15,17,18,19], cooking confidence [3,13,17,18,19], and nutrition knowledge [13,17,18]. Culinary interventions that have incorporated food and cooking skills alongside gardening, physical activity, or shared meal experiences and preparation activities have demonstrated further positive outcomes [15,20]. Improving both food and cooking skill levels contribute to improvements in diet quality [3], with some evidence that food skills are a better predictor of diet quality compared with cooking skills [1,4]. Cooking skills may also play a role in preparing and consuming foods consistent with sustainable nutrition principles, as limited cooking skills for plant-based foods is reported as a barrier to reducing meat consumption [21]. Furthermore, improving food agency, which is associated with food and cooking skills, can lead to greater cooking frequency, including more frequent cooking from scratch, and higher intake of vegetables [22]. This evidence highlights the important role of culinary education programs in enhancing participants’ food agency and both food and cooking skills.

However, not all studies report effects of culinary education on target health outcomes, and only one review in the field performed meta-analysis, finding no significant association between culinary education programs and anthropometric or cardiometabolic outcomes [15]. Research on the effects of culinary education programs has been limited by a range of factors including availability of valid tools for process and outcome evaluation, and the variable quality of other study design characteristics [3,13,15,16,17,18,19]. Culinary education research to date is further limited by insufficient reporting on the method of developing programs and how content is selected and prioritised [3,23]. Wolfson et al. reported that culinary education programs typically focus on “discrete mechanical tasks” [24], with little information provided about how program content and cooking tasks were selected for inclusion, and whether improving diet quality and health were considered [24]. There is a need for culinary education program developers to provide a rationale for food and cooking skill selection [24].

Box 1 **Definitions.**

Cooking skills: include food preparation techniques such as chopping, mixing, and heating [25,26] that may or may not require kitchen equipment. Cooking requires perceptual skills to understand how various foods react when manipulated and conceptual skills to understand how different food preparation techniques impact on the taste, colour, and texture of foods [25].

Food skills: are a distinct set of non-cooking skills where knowledge is applied to plan nutritious meals and snacks; select, acquire, and store ingredients; and dispose of food-related waste [27,28].

Food agency: is a framework for understanding the act of cooking within the myriad of factors that influence one’s ability both to obtain cooking skills and execute those skills within the contexts of one’s social, physical, and economic environments [24,29].

The Cooking Education (Cook-Ed^TM^) model was published to assist culinary education program providers with the complex task of designing, implementing, and evaluating programs that specifically aim to improve diet and health [30]. During the development of the Cook-Ed^TM^ model [30], a gap was identified in the availability of tools for culinary education program providers to assist them in selecting which food and cooking skills to teach within time-limited programs that aim to improve diet quality and health. Such a tool could help strengthen the evidence for food and cooking skill education programs, promote efficient use of program resources, and support development of programs to improve diet quality and health. 

The current study addresses this gap through development of the Cook-Ed^TM^ matrix to guide selection of food and cooking skills for inclusion in culinary education programs that target improved participant diet quality and health. This paper describes a modified e-Delphi process used to construct the matrix. The final Cook-Ed^TM^ matrix is provided in Table 1, and this paper also discusses its potential applications as an applied tool that is highly recommended to be used within the context of applying the Cook-Ed^TM^ model [30]. 

## 2. Materials and Methods

### Matrix Construction

The Cook-Ed^TM^ matrix was developed collaboratively by authors in Australia, Canada, Switzerland, United States, and the United Kingdom with consideration to the findings of the global review of FBDGs by Herforth et al. [31] to enhance its international relevance. Here the three-step process of developing the matrix and its adaptability for international use is described.

- *Step 1. Developing the Structure of the Matrix*

The final version of the Cook-Ed^TM^ matrix (Table 1) included 117 skill focus points spread across six sections: (1) Kitchen safety skills, (2) Food safety skills, (3) General food skills, (4) Food group specific food skills, (5) General cooking skills, (6) Food group specific cooking skills. As the key function of the matrix is to assist with improving the diet quality and health outcomes of culinary education program participants, sections four and six of the matrix include skill focus points categorised by common food groups identified in Herforth et al.’s review [31].

Herforth et al.’s review reported that over 90 countries have FBDGs developed for general populations, with 78 countries also having a “food guide” with graphic representations. However, many had caveats around the use of the FBDGs, or separate guidelines for people at a different life stage or people classified as not ‘healthy’. Across the food guides examined in the review, the most common food groups included: starchy staples, vegetables and fruits, protein sources (including meat, poultry, fish, eggs, legumes, nuts, and seeds), dairy and dairy alternatives, fats and oils, and foods and food components to limit. Therefore, columns within matrix sections four and six represent all of these food groups.

- *Step 2. Identifying and Mapping Culinary-Related Skills to Include in the Matrix*

Author RCA (a qualified chef, dietitian, and culinary nutrition researcher) reflected on her training and experience in kitchen and food safety, and reviewed consumer food safety guidelines [32] to draft the initial list of skill focus points in matrix sections one and two. Authors RCA and VAS (a dietitian and culinary nutrition researcher) then drafted a list of general food skill focus points, applicable to all food groups, in section three, and a list of food group-specific food skill focus points in section four based on food skills described by Fordyce-Voorham [27], McGowan et al. [3] and Lavelle et al. [33]. They also drafted a list of general cooking skill focus points in section five of the matrix and food group-specific cooking skill focus points in section six based on domestic cooking skills described by Raber et al. [34], McGowan et al. [3], Lavelle et al. [33], and Short [25,26]. In line with the purpose of the matrix, in sections three to six, only skills considered relevant to promoting diet quality and health were included. For example, healthier cooking methods such as steaming are included in the matrix, whereas less healthful methods such as deep frying are not included. Complex or specialised techniques that would not be necessary for domestic cooking e.g., sous vide, were also not included.

- *Step 3. Modified e-Delphi Process*

**Figure 1:**
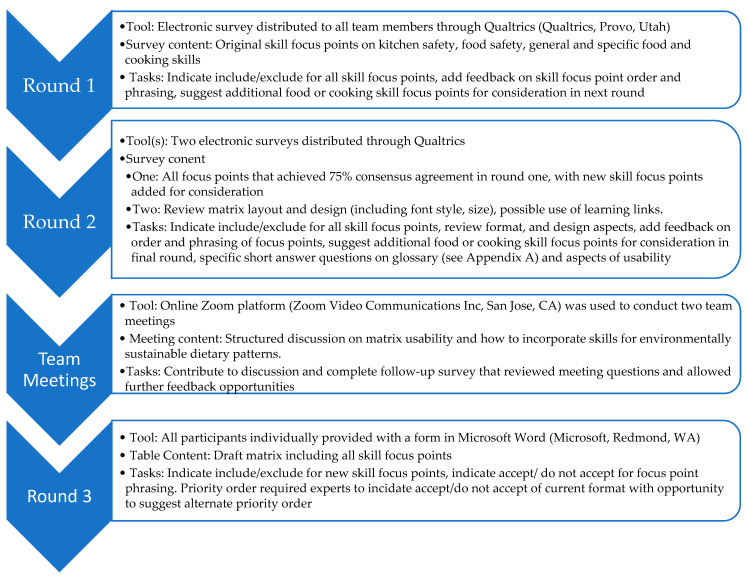
The modified e-Delphi process used in this study.

The modified e-Delphi process used in this study is shown in Figure 1. In each round, team members were asked to consider the study purpose when giving their responses. As per a traditional Delphi method, three structured feedback rounds were conducted collecting responses from all participants, blinded to each other’s responses within each round. A cut off agreement rate of 75% [35,36,37,38] was required for decisions about the matrix structure, skill focus point inclusion, order, and wording to be implemented in the matrix presented in the following round and final matrix. The modified aspect of this e-Delphi process [39] was the addition of a structured collaborative meeting in between rounds two and three that provided the opportunity for all authors to participate and share ideas (non-blinded). Several key e-Delphi studies within the field of health and nutrition [36,37,38,39] were also used to inform the modified e-Delphi methodology used in this study. Round one of the modified e-Delphi took place in September 2019. Due to the disruption of the COVID-19 pandemic, the next two rounds were postponed and completed between August and October 2021. 

Round one involved nine authors as participants and a fellow researcher from the University of Newcastle who could not continue to contribute due to changing work commitments (see acknowledgements). Six additional colleagues and collaborators joined the team from round two through to completion. In total, 16 team members (all are authors of this paper) completed rounds two and three. Team members have extensive experience and training in one or more of the following fields: nutrition (CEC, LC, SFV, VAS), dietetics (CEC, JS, KD, RCA, RG, SS, TJ, VAS), commercial cookery (JAW, RCA), cooking and food skill (culinary) education research and/or program development (all authors), behavioural and consumer sciences (FL, KvdH, MD, TB), public health (CEC, JAW, JS), education and curriculum review (LC, SFV, TJ), and occupational therapy (AR). 

Ethical considerations: Ethical approval was not needed for this research as the participants were the authors and acted in a consultation capacity. 

## 3. Results

The final matrix includes 117 skill focus points, including 5 kitchen safety skills, 6 food safety skills, 19 general food skills, 24 food group-specific food skills, 5 general food and cooking skills, and 58 food group specific food and cooking skills (Table 1). Working down the matrix, each column lists the order in which the skills would typically be performed when preparing food i.e., acquiring, transporting, storing, preparing, cooking, disposing, repurposing, or recycling food or its by-products. A glossary of terms used in the matrix is provided in Appendix A.

### 3.1. Modified e-Delphi Consensus and Refinement of Food and Cooking Skill Focus Points 

Table 2 details changes in the number of skill focus points in each section of the matrix during the modified e-Delphi process. Throughout the e-Delphi process, new skill focus points were created and some skill focus points were merged. There were 13 skill focus points that did not reach the ≥75% consensus required for inclusion, and these were removed. Skill focus points did not achieve consensus on the basis of: (1) skills were deemed as beyond what is required to achieve a healthy dietary pattern, (i.e., butterfly meats or prepare dough) or (2) concepts that required a high level of existing nutrition knowledge, which were therefore out of the scope of achieving a healthy dietary pattern in the general population (e.g., understanding the functional properties of foods). Comments made by the participants during round three of the modified e-Delphi further highlighted the need to refine skill focus points to contain a verb statement constructed as a learning objective. Using Blooms Taxonomy [40], the majority of skill focus points were rephrased as a learning objective with an embedded safety, food, or cooking skill. 

### 3.2. Modified e-Delphi Team Meetings 

While the initial focus of the matrix was to identify food and cooking skills necessary to achieve a healthy dietary pattern, as discussions progressed, the importance of including food and cooking skills to support sustainable dietary patterns for human and planetary health emerged [41,42]. This resulted in existing skill focus points (Table 1) being reviewed to incorporate sustainable nutrition principles where practical and relevant, e.g., emphasising the importance of and practical ways of improving legume consumption (skill focus point 4.3.4), the concept of recycle, reuse, and reduce (3.19), and in developing processes to use the complete food source (6.1.2). These skill focus points were then reviewed in round three of the e-Delphi for inclusion or exclusion and optimal phrasing.

### 3.3. Using the Cook-Ed^TM^ Matrix Together with the Cook-Ed^TM^ Model to Determine Priority Food and Cooking Skills

**Figure 2:**
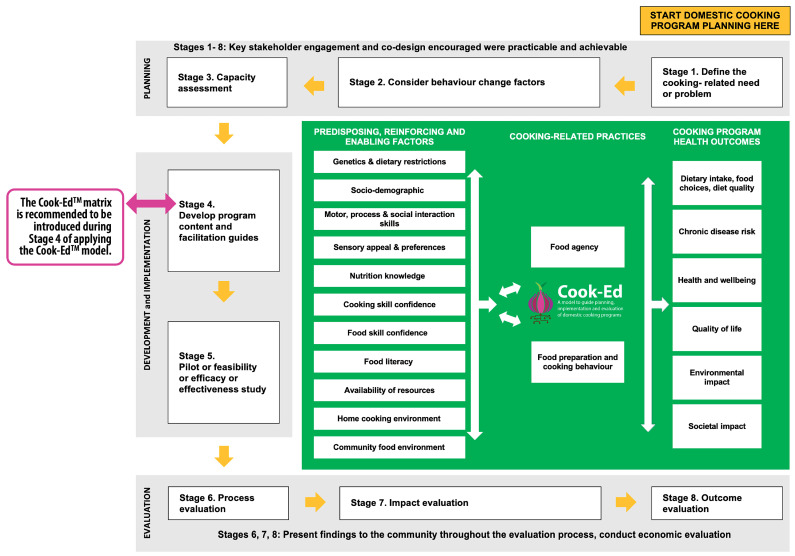
Illustration showing where to introduce the Cook-Ed^TM^ matrix when applying the Cook-Ed^TM^ model [30].

Where applicable, food and cooking skills specific to each of the common food groups are outlined in sections four and six of the matrix to ensure the necessary skills required to select, prepare, and ultimately consume a wide variety of foods from each of these groups can be achieved. While the matrix can be used on its own as an applied programming tool to guide content and learning materials, it is highly recommended that program providers use it within the context of applying the Cook-Ed^TM^ model [30] (as shown in Figure 2). The Cook-Ed^TM^ model has been created to assist program providers in tailoring culinary education programs to the needs of specific groups and guide them through all steps of program creation from conception and development to evaluation [30]. The selection and structure of culinary education program activities, such as food and cooking skill instruction, should align with the program aims and objectives as well as participant’s learning goals and needs [43,44]. Once program aims and objectives have been defined using the model (see Cook-Ed^TM^ model [30] Stage 4—“Develop program content and facilitation guides”), culinary education program providers can use the matrix (Table 1) to select and prioritise food and cooking skills to teach based on the needs and characteristics of the target audience and information gathered in program planning (see Cook-Ed^TM^ model [30] Stages 1 to 3—“Define the cooking-related need or problem”, “Consider behavior change factors”, and “Capacity assessment”). 

Health and safety principles should underpin food and cooking skill education programs to any audience and be a common thread integrated throughout the program. These are listed within section one and two of the Cook-Ed^TM^ matrix (Table 1). Throughout a cooking program, participants should receive appropriate information about health and safety, applicable to the demonstration kitchen and also the home setting where learned skills will be applied. This may include information on the safe handling of knives, electrical equipment, hot surfaces, slip or trip hazards in the kitchen, and appropriate kitchen attire. This is in addition to general food safety knowledge and practices to minimise microbial and other contamination of food. 

Program providers without nutrition and dietetic expertise are encouraged to consult with such qualified professionals in the planning phases to ensure that program content aligns with current dietary advice and nutrition principles.

When determining skills to include, life stage, cognitive and motor skills of participants also need to be considered, e.g., culinary education programs for younger children need to teach food and cooking skills that are developmentally appropriate [45]. For people with cognitive and/or physical impairments, the demands of the skills selected need to consider an individual’s capacity to perform the skill and the availability of helpful modifications (e.g., assistive technology). Consultation with an occupational therapist is suggested to support efficient and effective skill development and/or adaptation to the environment or activity to enable participant engagement.

The Cook-Ed^TM^ matrix has been designed to support practical application of learning theory by program providers. The matrix assists with the selection of appropriate activities and can therefore support matching of both food and cooking skill development needs with the current skill levels of program participants. Evaluation data gathered may also be used to modify future programs in an iterative manner.

### 3.4. Consdering Appropriate Skill Level of Cook-Ed^TM^ Matrix Items 

To enhance usability of the Cook-Ed^TM^ matrix, the concept of tiered learning opportunities (See Box 2) for some skill focus points was raised in the e-Delphi. For example, skills focus points could be further broken down into basic, intermediate, and advanced skills. The basic level is suitable to achieve a healthy dietary pattern, with intermediate and advanced levels offering enhanced skills to expand food and cooking skill development opportunities. This concept would allow program providers to select the level of the skill focus point that is best suited to the abilities and needs of their participants and adapt teaching as their skills increase, allowing them to build on skills previously acquired.

Box 2 **Example of a tiered learning opportunity for skills focus point 6.3.1.**

Original: prepare legumes, and minimally processed/whole food alternatives

Basic: identify low/no sodium tin/canned legume varieties, drain, and rinse for use

Advanced: purchase dried legumes and prepare using pressure cooker to reduce cooking time, freeze excess for use in other dishes.

## 4. Discussion

The Cook-Ed^TM^ matrix is a comprehensive set of safety, food, and cooking skills specific to common food groups in FBDGs. To our knowledge, the matrix is the first tool available, generated through expert consensus, to guide researchers and culinary education program providers in selecting skills to improve diet and health outcomes. The skill focus points aim to promote development of skills required to achieve healthy dietary patterns that align with FBDGs for a general population and incorporate sustainable nutrition principles. It is recommended that the Cook-Ed^TM^ matrix be used in the context of applying the Cook-Ed^TM^ model [30], as illustrated in Figure 2, so that it guides culinary education program providers to select the most suitable skill focus points based on participants’ available resources and needs.

Limitations of cooking research to date include weak study designs, a high degree of heterogeneity in outcome measures and study populations, and poor reporting of program development activities, including selection of program content [3,16,17,18,19]. When used together (Figure 2), the Cook-Ed^TM^ model [30] and the Cook-Ed^TM^ matrix (Table 1) provides researchers and culinary education program providers with resources to strengthen the evidence for culinary nutrition education programs and their influence on diet quality and health. 

Consideration of other factors influencing cooking behaviour should be recognised. Healthy cooking behaviour is complex, and a myriad of personal, socioeconomic, cultural, and environmental factors can interact to influence cooking behaviour and diet quality [4,5]. Factors other than food and cooking skills, such as socio-demographic characteristics, nutrition knowledge, and psychological wellbeing are key influences on diet quality [4]. In a nationally representative sample of adults in the USA, Wolfson et al., [6] reported that cooking frequency does not influence diet quality equally across socio-economic groups, suggesting additional factors such as food provision may be more pertinent considerations for culinary nutrition education program providers when working with different groups. As recommended in the Cook-Ed^TM^ model [30], conducting an assessment of these factors before developing program content, and iteratively through program implementation, can inform education sessions focused on highest priority skills. Examples of prioritising skills in a culinary nutrition education program after assessment of the target audience can be found in Table 3. 

Complimentary activities such as shared meal preparation, sitting down to a shared meal at the close of a practical session, and taste testing may be considered, and are frequently associated with, positive outcomes [46,47,48,49]. These can enrich culinary learning programs and enhance learning experiences by encouraging group discussion, family food preparation and meal planning discussion beyond the program and provide participants with opportunities to try new recipes and unfamiliar foods and flavours [46,48,49]. Developing an appreciation of new flavours, tastes and foods, and increased preference for fruit and vegetables can support dietary intakes that align more closely with FBDGs [46,48]. Similarly, program providers may consider other social and physical activities, such as gardening, grocery store tours, or physical activity sessions that can enhance program outcomes [15].

Incorporating sustainable nutrition principles was not an a priori aim of the matrix. However, it was an important consideration raised by participants during the e-Delphi process who recognize that culinary education researchers, program providers, and consumers all have a key role to play in achieving environmentally sustainable nutrition goals that can also be compatible with achieving higher dietary quality and favourable health outcomes [42,50]. Informed by the growing evidence on healthy diets from sustainable food systems, the e-Delphi participants acknowledged that foods consistent with sustainable nutrition principles (e.g., unprocessed plant-based food) can require greater time, effort, and skill to prepare, and they must taste good and be culturally appropriate [42,50]. With the current developments towards sustainable nutrition, the complexity of food skills to be taught in interventions is increasing substantially, and therefore sustainable nutrition principles were considered an important element to consider when developing skill focus points in the matrix. 

The Cook-Ed^TM^ matrix may have other applications beyond dedicated culinary education programs. For example, many of the food skill components of the matrix could be used to guide the content of nutrition education sessions in health-related programs (e.g., in chronic disease prevention or treatment programs reviewing local nutrition recommendations for different stages of life and health needs, or investigating the nutrient profiles of each core food group, their functions, and roles), which may not always have the facilities, resources, or time allocation to practically teach cooking skills). Other examples include learning to recognise key nutrition and culinary terms, planning a menu to meet personal and household needs, and accompanying shopping/grocery list. Furthermore, with the onset of the COVID-19 pandemic, a transition to virtual education modes to deliver culinary education programs has been more common with programs facilitated outside the traditional kitchen space [51,52]. 

A strength of this study is that the authors contributing to the development of the matrix are an international team, but it needs to be highlighted that this expertise is focused across countries with similar food and cooking cultural requirements. The Cook-Ed^TM^ matrix has broad international relevance, but culinary education providers should consider the items in the matrix within the context of their own FBDGs, food and cooking culture and practices, and food availability and adapt the matrix accordingly. Additional food and cooking skills may need to be considered for the matrix to be applied to programs for other cultural groups and countries with eating patterns other than a Western diet. Similarly, additional food and cooking skills may need to be considered for non-domestic culinary education programs (e.g., commercial cookery programs), or programs where skills to improve diet quality and health are not the primary aim. A limitation of the matrix is that it is not a comprehensive list of all food and cooking skills that could be included in culinary programs 

A further strength is that the design of the matrix and skill focus point selection process, via a modified e-Delphi process with three rounds, permitted independent and deep analysis to develop the final skill focus points shown in the matrix in Table 1. It is acknowledged that while the use of the Herforth et al review of FBDG provided a structured approach for linking the matrix learning objectives with dietary quality outcomes, the approach to incorporating sustainable nutrition principles was less structured [31].

## 5. Conclusions

The Cook-Ed^TM^ matrix presented is an evidence-based applied tool to assist in the selection and prioritisation of food and cooking skills for inclusion in culinary nutrition education programs to improve diet quality and health of participants. The matrix can be used in a variety of global settings by adapting outcomes to meet country-specific FBDGs. By detailing the process of developing the matrix and publishing it here as a freely available tool in an open access journal, cooking program providers in a variety of settings will be able to use the Cook-Ed^TM^ matrix as a program development tool. To assist with tracking the application and impact of the Cook-Ed^TM^ matrix, we encourage users to acknowledge when and how the matrix was used in their projects. When used together with the Cook-Ed^TM^ model [30], the Cook-Ed^TM^ matrix supports program providers in selecting and prioritising food and cooking skills relevant to their participant group based on program goals, nutrition recommendations for different life stages, and participant skill development needs and preferences. Further research is needed to examine the application of the matrix as an applied tool to guide program content development across a wide range of settings and target groups. 

## Figures and Tables

**Table 1:** The Cook-Ed^TM^ matrix to guide skill selection in culinary education programs that target improved diet quality and health.

**1. Kitchen safety skills**				
1.1 Demonstrate familiarity with kitchen layout, equipment, and appliances 1.2 Demonstrate awareness while working in the kitchen and clear communication practices when working with others 1.3 Demonstrate appropriate personal hygiene 1.4 Implement correct procedures to maintain clean kitchen space, equipment, and utensils 1.5 Implement an ordered and functional workspace				
**2. Food safety skills**				
2.1 Assess expiry information on packaged foods to select items for immediate use, or with sufficient storage life for pantry and future use 2.2 Develop visual and olfactory senses to identify when food may be spoiled (no longer edible) 2.3 Recognise key food allergens 2.4 Apply correct transport, storage, and reheating food practices to minimise spoilage, microbial contamination, or cross-contamination 2.5 Implement strategies to avoid cross-contamination when cooking 2.6 Implement safe food preparation practices				
**3. General food skills applicable to all food groups listed below**				
3.1 Review local nutrition recommendations, where provided, for different stages of life, gender, and health needs e.g., diabetes, hypertension 3.2 Investigate the nutrient profiles of each core food group, their functions, and roles 3.3 Recognise and understand commonly used nutrition terms 3.4 Recognise and understand commonly used culinary terms 3.5 Assess the need for variety to support a healthy dietary pattern 3.6 Recognise culinary terms of measurement and apply common methods of conversion 3.7 Prepare a dish/meal using a recipe 3.8 Plan a menu for a set period that meets household dietary needs, considering ecological footprint, available resources, and food budget 3.9 Plan a grocery/shopping list based on a menu plan for a set period 3.10 List common staple ingredients and describe appropriate storage methods for these foods			3.11 Assess food products using food label information and price to select most nutritious options that are compatible with sustainable practices and/or resources available 3.12 Identify sustainable food selection and preparation practices 3.13 Use ingredient substitutions for recipes when food items are unavailable or unsuitable 3.14 Use leftover ingredients to make another meal/dish 3.15 Implement culinary short-cuts to prepare a nutritious meal/dish when time is limited to suit skill level or reduce the work of cooking 3.16 Select suitable recipes for large group sizes, batch cooking for freezing, and/or for use in multiple meals 3.17 Prepare a meal with limited ingredients or resources 3.18 Develop planning and kitchen set up processes (mise en place) before meal preparation to enhance efficiency 3.19 Recognise correct reduce, reuse, and recycle processes of food and non-food kitchen waste	
**4. Food group specific food skills**				
**Vegetables & Fruit**	**Grains**	**Meat & Alternatives**	**Dairy & Alternatives**	**Extras**
4.1.1 Select in season unpackaged produce or minimally packaged produce or low sodium/low sugar packaged alternatives considering price, availability, and sustainable food practices 4.1.2 Identify veg or fruit with short vs. long storage life, purchase and use accordingly to promote diet variety and minimise wastage 4.1.3 Know when and how to clean/wash produce 4.1.4 Apply appropriate storage techniques for stage of ripeness and nutrient retention 4.1.5 Identify techniques and suitable uses for food that is bruised, imperfect, or approaching end of life but still safe for consumption 4.1.6 Identify ways to include different types of veg into snacks and each meal type of the day (e.g., B, L, D) 4.1.7 Modify recipes to include more veg	4.2.1 Identify and select wholegrain and wholegrain based products 4.2.2 Identify grain foods for multiple purposes and to increase wholegrain intake and variety 4.2.3 Know how to use when approaching end of life but still safe for consumption 4.2.4 Modify recipes to increase fibre	4.3.1 Identify and select minimally processed/wholefood meat alternatives to create a variety of plant-based meals 4.3.2 Identify and select lean meats, low sodium and minimally processed/wholefood meat, and meat alternatives 4.3.3 Select recipes that utilise a range of cooking techniques to prepare different cuts of meat, fish varieties, or alternatives considering budget, nutrition, and ecological footprint 4.3.4 Identify a variety of legumes and corresponding preparation and cooking methods 4.3.5 Identify and know how to select eggs or suitable egg alternatives for different purposes and know suitable recipe substitutions 4.3.6 Modify recipes to use lower salt and/or lower saturated fat meat and alternatives	4.4.1 Recognise core vs. extras/non-core dairy or alternatives products 4.4.2 Review the nutritional composition of plant-based milk alternatives to select the most suitable to meet nutritional needs and requirements 4.4.3 Select shelf stable varieties if access to fresh varieties or suitably healthier options is limited 4.4.4 Modify recipes to use lower salt and fat reduced products	4.5.1 Review packaging information to identify extra/non-core foods and/or ingredients and select better alternatives 4.5.2 Modify convenience foods to increase nutrition content 4.5.3 Modify recipes to use or incorporate more core group foods and to replace non-core food items
**5. General cooking skills applicable to all food groups listed below**				
5.1 Know what cooking methods are suitable to retain nutrients and flavour 5.2 Select healthier oils in suitable amounts to match recipe style 5.3 Develop dishes that add flavour using herbs, spices, and acidic foods as a way of minimising or as an alternative to salt 5.4 Create dishes without a recipe from available resources 5.5 Investigate what flavours, textures and foods complement each other				
**6. Food group specific cooking skills**				
**Vegetables & Fruit**	**Grains**	**Meat & Alternatives**	**Dairy & Alternatives**	**Extras**
6.1.1 Demonstrate how to properly wash or clean 6.1.2 Develop processes to use the complete food source (where appropriate) to increase food variety and reduce food waste 6.1.3 Peel (or not ) 6.1.4 Pick/tear leaves 6.1.5 Slice, dice/cube ^µ^ 6.1.6 Grate ^µ^ 6.1.7 Boil and simmer 6.1.8 Microwave to retain nutrients 6.1.9 Pan fry/shallow fry, stir fry, sauté 6.1.10 Stew/slow cook 6.1.11 Blend to make a soup, puree, or sauce using available equipment ^µ^ 6.1.12 Grill 6.1.13 Roast 6.1.14 Steam 6.1.15 Poach/Blanch 6.1.16 Prepare a variety of simple cold or hot veg dishes without a recipe 6.1.17 Identify required cooking times for individual veg or as part of a composite meal 6.1.18 Prepare a stock using saved veg peelings 6.1.19 Prepare a fruit-based sauce with no or minimal added sugar 6.1.20 Use a pressure cooker	6.2.1 Weigh and measure dry ingredients 6.2.2 Identify grains that need to soak and use appropriate timing 6.2.3 Microwave 6.2.4 Boil & simmer 6.2.5 Absorption method 6.2.6 Pan fry/shallow fry 6.2.7 Knead dough 6.2.8 Steam 6.2.9 Prepare wholegrain snacks and dishes for each meal of the day (e.g., B, L, D) 6.2.10 Prepare healthier baked products from scratch	6.3.1 Prepare legumes and minimally processed/wholefood alternatives 6.3.2 sup>∙ Slice, dice/cube ^µ^ 6.3.3 Prepare meat cuts for cooking by trimming off excess fat ^µ^ 6.3.4 Prepare meat or seafood-based stock using saved bones, skin, or fillet from fish, chicken, or beef 6.3.5 Prepare eggs 6.3.6 Preparing egg alternatives for different purposes 6.3.7 Pan fry, shallow fry, stir fry, sauté 6.3.8 Boil and simmer 6.3.9 Stew/slow cook 6.3.10 Grill 6.3.11 Steam 6.3.12 Poach 6.3.13 Blend to make a soup, puree, or sauce using available equipment 6.3.14 Roast 6.3.15 Cook meat, poultry, fish, legumes, and meat alternatives to correct temperature, safe for consumption and palatability 6.3.16 Prepare an egg-based (or egg alternatives) dish 6.3.17 Use nuts/seeds in a variety of dishes/snacks for non-allergic participants to increase nutrient value or as a suitable plant-protein substitute with other ingredients 6.3.18 Prepare healthier meat-based sauces from scratch 6.3.19 Prepare healthier marinades 6.3.20 Use a pressure cooker	6.4.1 Weigh and measure liquids, semi-solid and solid food 6.4.2 Grate ^µ^ 6.4.3 Apply heat 6.4.4 Prepare healthier dairy or dairy alternatives-based sauces and dressings	6.5.1 Identify and prepare recipes where high saturated fat ingredients can be swapped for monounsaturated and polyunsaturated fat alternatives 6.5.2 Prepare a typical convenience food using core foods to increase nutritional content 6.5.3 Prepare a healthy beverage from fruit, veg, or dairy/alternatives ingredients 6.5.4 Prepare healthy snacks using a combination of nuts/seeds, grains, fruit, dairy/or alternatives
**Abbreviations/key:** All section headings and sub-headings appear as bolded text, B—breakfast, L—lunch, D—dinner, nutr—nutrient/nutritious/nutritional, w/out—without, info—information, veg—vegetables, vegetable. + ‘Use only sometimes and in small amounts’ and ‘Use in small amounts’ as per Australian Guide to Healthy Eating [8], µ include knife/sharps safety training				
**How to use the Cook-Ed^TM^ matrix**				
The Cook-Ed^TM^ matrix is a comprehensive table of skills for consideration in cooking education programs that aim to primarily improve diet quality and health. The skills represent those required to prepare basic food groups so that eating patterns align with common food-based dietary guidelines (FBDG) in a general population [31]. The Cook-Ed^TM^ matrix could be adapted to different populations and different FBDGs. Working across the matrix from left to right are common food groups. Working down each section, skill focus points are ordered in the Cook-Ed^TM^ matrix in the sequence that aligns with typical food preparation i.e., acquiring, transporting, storing, preparing, cooking, disposing, re-purposing, or recycling food or its by-products. To tailor culinary education programs to the precise needs of specific groups, program developers are advised to use the Cook-Ed^TM^ matrix with the Cook-Ed^TM^ model [30] to identify which skills are most relevant and re-arrange the matrix based on factors that have been identified in the planning or evaluation stages of program development. This might include the dietary needs of the specific group or the program aims.				

**Table 2:** Kitchen safety, food safety, food skill, and cooking skill focus points: Selection and categorisation throughout modified e-Delphi rounds.

	Matrix Section	Food Group	Focus Points (n)			
Year			2019		2021	
e-Delphi Round			Original	1	2	3
	1. Kitchen Safety Skills		2	5	5	5
	2. Food Safety Skills		9	6	6	6
	3. General Food Skills		20	17	20	19
	4. Food Group Specific Food Skills	Vegetables	7	7	9	7
		Fruit	6	6	*	*
		Grains	4	5	5	4
		Meat and Alternatives	4	5	7	6
		Dairy and Alternatives	5	3	4	4
		Extras	2	3	3	3
	5. General Cooking Skills		5	5	6	5
	6. Food Group Specific Cooking Skills	Vegetables	20	20	21	20
		Fruit	9	8	*	*
		Grains	11	12	10	10
		Meat and Alternatives	23	22	20	20
		Dairy and Alternatives	7	6	4	4
		Extras	4	4	4	4
Total			138	134	124	117
Team members participating (n)			2	7	15	15
Tools used in e-Delphi round				Qualtrics Survey	Qualtrics Survey	Structured Table

* Fruit merged with Vegetables subgroup from round 2.

**Table 3:** Example of prioritising skills in a culinary nutrition education program.

Participant Context	Do not Prioritise	Do Prioritise
Limited access to fresh produce due to finances, availability, or capacity to safely store food	Preparation skills of mainly fresh vegetables and fruit Recipes with expensive ingredients, batch cooking for freezing, and/or for use in multiple meals	Food and cooking skills for frozen, canned, and/or identify long-storage shelf life vegetables (e.g., cabbage) Preparation of single portion meals using econonomical ingredients
No access to a blender	Blended soups, puree, or sauce	Soups or dips that remain texturally and visually appealing when mashed with a fork and served chunky Assess food products using food label information and price to select most nutritious soup, puree, or sauce options that are compatible with sustainable practices and/or resources available
Young children not yet able to use knives and hot cooking equipment independently	Meals and snacks that require extensive cutting with large sharp knives (e.g., pumpkin) and/or use of heat	Meal and snack assembly skillsSoft food items that can be easily cut with appropriate knives (e.g., banana, mushrooms)

## Data Availability

The complete dataset generated from this study are available on request from the corresponding author.

## Appendix A

**Table A1:** Glossary of key terms within the Cook-Ed^TM^ matrix.

Term	Definition
**Kitchen & Food Safety**	
Cross-contamination	Unintended movement of micro-organisms, contaminants, or allergens from between foods e.g., from raw food to cooked food [53].
Microbial contamination	Unintended introduction of potentially harmful microorganisms (e.g., bacteria, mould, fungi, yeast) into food.
Personal hygiene	The practice of maintaining a standard of cleanliness of one’s body. Personal hygiene required for food preparation can include hand and body washing, cough and sneeze etiquette, maintenance of hair and nails, clothing.
**Food & Nutrition Terminology**	
Beans	A type of legume, examples include red kidney beans, black beans, borlotti beans.
Chickpeas	A type of legume.
Convenience food	Food that requires little preparation or cooking prior to consumption. Often refers to commercially prepared food such as TV dinners, ready-meals, frozen meals.
Cooking skills	Include a range of food preparation techniques such as chopping, mixing, and heating [25,26] that may or may not require kitchen equipment. Cooking requires perceptual skills to understand how various foods react when manipulated and conceptual skills to understand how different food preparation techniques impact on the taste, colour, and texture of foods [25].
Core foods	The Australian Dietary Guidelines definition “foods that form the basis of a healthy diet, based on or developed with reference to recommended daily intakes (RDIs)” [8].
Core food groups (within the Cook-Ed^TM^ matrix)	Vegetables and Fruits, Grains, Meat and Alternatives (e.g., legumes, nuts, seeds, tofu), Dairy and Alternatives.
Dietary fibre	Edible part of plant food that resists digestion in the small intestine and may be fermented to varying degrees in the large intestine. Includes soluble and insoluble fibre and resistant starches.
Dietary pattern	Refers to the variety, amount, and combination of food and drinks in the diet and the frequency with which they are habitually consumed.
Fats & Oils	Edible fats and oils occurring naturally in food, used in food manufacturing or cooking. May also be referred to as dietary fat. Dietary fats can be classified as saturated fat, trans fat, polyunsaturated fat, and monounsaturated fat. Edible fats and oils typically contain a combination of the different dietary fat.
Food-based dietary guidelines	The Food and Agriculture Organization of the United Nations definition (also known as dietary guidelines) are intended to establish a basis for public food and nutrition, health and agricultural policies, and nutrition education programmes to foster healthy eating habits and lifestyles. They provide advice on foods, food groups, and dietary patterns to provide the required nutrients to the general public to promote overall health and prevent chronic diseases*”* [7].
Food skills	Include meal planning, shopping, budgeting, resourcefulness, and interpreting food labels and nutrition information panels [27,33]. Lavelle et al. 2019 used psychometric testing to delineate food skills as a distinct set of non-cooking skills that enable individuals to apply knowledge about food to then prepare meals and snacks that are nutritionally appropriate within the available resources [33].
Food waste	The Food and Agricultural Organization definition “the decrease in quantity or quality of food resulting from decisions and actions made by retailers, food service providers and consumers” [54].
Grains	Commonly referred to as cereals or cereal grains and which are the edible seeds of specific grasses [8].
Legumes	Plant in the Leguminosae (Fabeceae) family. The term legume may also be used to refer to the edible seed or pod (e.g., beans, lentils, peas, and chickpeas). Legumes come in a variety of shapes, sizes, and colours.
Lentils	A type of legume, examples include yellow lentils, brown lentils, red lentils.
Meat alternatives	Can include a range of wholefood items such as nuts, seeds, legumes and mushrooms, or minimally processed foods made from combinations of these.
Menu plan	A detailed list of dishes and/or recipes for a specific meal, day, or week.
Minimally processed	NOVA classification definition “natural foods altered by methods that include removal of inedible or unwanted parts, and also processes that include drying, crushing, grinding, powdering, fractioning, filtering, roasting, boiling, non-alcoholic fermentation, pasteurisation, chilling, freezing, placing in containers, and vacuum packaging…methods and processes … designed to preserve natural foods, to make them suitable for storage, or else to make them safe or edible or more pleasant to consume” [55].
Non-core food	Foods that do not fit within the definition of ‘core foods’ (refer to core foods).
Non-core food group (within the Cook-Ed^TM^ matrix)	Extras (also called energy dense, nutrient poor foods, discretionary or junk).
Nutrient dense foods	A good source of essential macro and micronutrients.
Peas	A type of legume, examples include chickpeas, black-eyed peas, split peas.
Plant-based	A meal or dietary pattern that focuses on including mostly core foods that come from vegetable, fruit, nuts, seeds, legumes, and wholegrain groups.
Pulse	The edible, dried seed of a legume (e.g., beans, lentils, peas, chickpeas). The term legume is used to describe pulses within the matrix.
Processed products	Made by adding salt, oils, sugar or items used to prepare and/or season food. Using preservation methods such as canning, bottling, and in some cases using non-alcoholic fermentation processes [55].
Shelf life	The expected length of time a food will maintain its best quality [53].
Shelf stable	Does not require refrigeration.
Staple food	Food item(s) that are eaten frequently and form the basic components of a usual dietary pattern [53].
Storage life	Time in which a food item can be safely kept in the fridge, freezer, or pantry to maintain quality and remain edible.
Sustainable eating	Selecting foods that are healthful for the environment and that support human health.
Vegan	A meal or dietary pattern that includes only foods from plant-based origin.
Vegetarian	A meal or dish that focuses on including mostly core foods that come from vegetable, fruit, nuts, seeds, legumes, and wholegrain foods with variation that can include some dairy, seafood, and eggs.
Ultra-processed	NOVA classification definition “formulations of ingredients, mostly of exclusive industrial use, made by a series of industrial processes, many require sophisticated equipment and technology…colours, flavours, emulsifiers and other additives … to make the product palatable or hyper-palatable*”* [55].
**Culinary terms**	
Absorption method	Wholegrains like rice or quinoa, place in cool water, bring the water to the boil, simmer for a short period and then turn off heat and cover with pot lid for the remainder of the cooking time to allow grain to absorb liquid and finishing cooking for a drier, fluffy result.
Baking	Cook food in dry heat in an oven.
Blanch	To subject food to boiling water by plunging into boiling water and removing after a few seconds.
Blend (puree)	Using a mini blender/stick blender/bar mix or hot blender (e.g., Thermomix^TM^) to produce a finely mashed, smooth, or liquid consistency.
Boil	Cook food submerged in a boiling liquid, or food being cooked at boiling point.
Dice/cube	To cut even pieces in the rough shape of a dice or cube, size of dice/cube dependent upon the dish being prepared.
Grate	Using a vegetable/box grater, run the food item down and up or along the grater, being mindful of having the grater set securely on a cutting board or large plate/bowl and to keep fingers at a safe distance.
Grill	Cook food by radiant heat. May also be referred to as broiling or barbequing.
Pan fry	Cook food in a small amount of fat/oil. May also be referred to as shallow frying or sauté.
Poach	Cook food in a liquid that is below boiling point.
Roast	Cook food in dry heat in the presence of fat/oil.
Sauté	Cook food in a small amount of fat/oil. May also be referred to as pan frying or sauté or gently fry.
Scratch cooking	Cook food from raw or minimally processed ingredients.
Simmer	Cook food submerged in a liquid that is just below boiling point but bubbling.
Slice	To cut thin pieces either along or across the food item depending on the dish being prepared.
Stew/Slow cook	Cook food at a long temperature for an extended period of time in a sufficient amount of liquid; the food and cooking liquid are typically served together.
Steam	Cook food in steam/vapour.
Shallow fry	Cook food in a small amount of fat/oil. May also be referred to as pan frying or sauté.
Stir fry	Cook food in a small amount of oil, often at a high heat for a short period of time while stirring constantly. Stir frying is often performed in a wok (bowl shaped pan).
**Key:** the section headings and subheadings appear as bolded text	
